# Paratexts on a social network site and their relevance in the production of meaning—Results of a qualitative investigation of Twitter-Feeds

**DOI:** 10.1371/journal.pone.0238765

**Published:** 2020-09-18

**Authors:** Matthias Völcker

**Affiliations:** Institute for Educational Science, Georg-August-University Göttingen, Göttingen, Germany; Catholic University of the North, CHILE

## Abstract

In this paper, paratexts as a component of developmental and marketing processes of movie-productions on Social Network Sites (SNS) are investigated. Paratexts are phenomena that prepare and accompany the reading and interpretation of texts/movies. First, a brief introduction into a complex and ambivalent state of research on paratexts will be given. Then the paper presents the results of a qualitative study, which was realized with the help of Grounded Theory, where marketing-paratexts of movie feeds on Twitter are at the center of the investigation. The research shows that movie-studios are manifoldly active on Social Network Sites and fall back on different paratextual materials that are placed around the medium as an interpretative perimeter. The associated activities are also characteristics of complex interactive processes. In this, recipients are actively involved. Producers/Production-Studios attempt to establish a relationship, whereby Social Network Sites are used as distributional and interactive platforms and as a vital part for preparing and developing a story.

## Introduction

On 03/30/2017 the online spin-off of the German-language news magazine DER SPIEGEL reported in an article about the viral success of a teaser trailer for the movie *IT* (2017, Andrés Muschietti) and its as extraordinary designated success on Social Network Sites. What was uncommon about this was that such a ‘by-product' of popular culture–a teaser for a horror movie–became part of the regular reporting. However, the article implicitly pointed to the increased significance of such accompanying phenomena in a networked society and their functions in yielding attention, curiosity, hype and occasions for meaning production [1]. The teaser reached traffic hits on Social Network Sites and User Generated Content Sites [SNS and UGC] that are exceptionally high, not only for a horror movie. On Facebook alone, the teaser was viewed over 85 million times during the first 24 hours of its release.

In scientific discourse, such media by-products are called paratexts. Paratexts consist “empirically of a manifold number [of] practices and discourses” [2; p. 10], which prepare and accompany the readings of a text as companion pieces. They surround “texts, audiences, and industry, as organic and naturally occurring a part of our mediated environment as are movies and television themselves” [3; p. 23]. In contrast to Genette, who “sees two things: the text and the paratext […, where] the paratext is always outside the text, and it`s likely always secondary”, Brookey & Gray argue “that paratexts are intrinsic parts of the text as social and cultural unit” [4; p. 102, 5]. In their functionality, they are described as “a way of getting into a text” [3; p. 149]. Paratexts can be differentiated: They are produced and distributed firstly from the perspective of producers, and then primarily professionally and under marketing considerations [marketing-paratexts]. On the other hand, however, paratexts also arise as a result of an active as well as creative appropriation and interpretation as individual readings, for example, by loyal and at the same time passionately dedicated fans. Their inventive, innovative, as well as resistant interpretations can be found in fan-fictions, fan-posters, fan-songs or fan-trailers, as well as in preparing future text receptions, for example via the reception and discussion of spoilers and leaks [audience-created-paratexts] [6–8].

Particularly, marketing-paratexts are an opportunity for audiences to obtain first insights into a cinematic narrative, to experience visual characteristics of a movie, but also characters and plot components. Paratexts offer and create frames through which recipients can make sense of what a text is or could be for them. They are part of an interpretative scope that operates under commercial and marketing considerations whereby production studios seek to establish bonds with recipients and to offer opportunities for meaning-production as a result of interactions and establishing relationships. This raises the question of how such meanings, e.g. as via Social Network Sites (SNS), are offered, and “how meaning begins and where texts come from” [3; p. 47].

This article focuses on how these kinds of paratexts establish offers for meanings and regulate the relationship between texts, audiences, and industry/producers. The paper analyzes this phenomenon from different perspectives. In the following two chapters, the state of research on paratexts and their meanings as a facet of textual framing are presented (2). On the one hand, marketing-paratexts focus on the financial exploitation of a medial consumer product. On the other, the related activities, particularly on SNS, are aimed at the dissemination of content and the preparation of texts/movies. Recipients become a vital part in these procedural activities, with emotional engagement, interest, and curiosity intended to encourage involvement with a text (3). In this sense, “promotion […] is not only the commercial act of selling but also of advancing and developing a text” [3; p. 5]. Paratexts provide meanings that are fluid and negotiated discursively [9; p. 99]. Using the example of an investigation of Twitter feeds, the essay then examines with the help of qualitative analysis the functions of marketing-paratexts, reconstructing the efforts and activities undertaken by moviemakers and producers to arouse interest, curiosity, hype and emotional bonds (4+5).

## Paratexts and their relevance in the movie production process

Paratexts are textual fragments that surround a text/movie, but cannot be seen as the text itself [3,10–13]. They fill the gaps between audiences, the industry, and the text. Paratexts “include everything from book covers to movie posters, ads in magazines, to trailers, critical reviews to alternate reality games, special edition DVDs to spinoff toys, roadside billboards to fan-fiction and film” [14; p. 94] as well as previews, reviews, making-ofs, production diaries, bonus materials, deleted or extended/alternate scenes, commentary tracks and even word of mouth, which frame our interpretation[s] of a movie [15,16]. A text/movie "is not a finished production, but a continuous productivity” [3; p. 7], with paratexts offering opportunities for orientation [17; p. 165] and thus are a vital part of the storytelling. What matters is how paratexts contribute to audiences' meaning-making and their relevance as part of the 'storyfication'.

Paratexts are accompanying texts, “which transform and condition how the audience interprets the main text” [18; p. 25]. Both audience-created, as well as marketing-paratexts, operate as connecting links between text and recipients. They focus the text, but can also include textual extensions and offer specific, intertextually appropriation opportunities. Paratexts accompany the path to a movie, but can also be of relevance following its release [3; p. 24, 16], just as they become relevant in the moment of the movie reception in the opening and end credits [5,19]. Gray differentiates paratexts further into two categories: entryway paratexts and in medias res paratexts. Entryway paratexts are texts “that grab the viewer before he or she reaches the text and try to control the viewer’s entrance to the text”. In contrast in medias res paratexts are texts “that flow between the gaps of textual exhibition, or that come to us ‘during’ or ‘after’ viewing, working to police certain reading strategies” [3; p. 23, 4].

While movie poster, teaser, and trailer in their paratextual functionality are today still considered part of the framework program of a visit to the movies [20], their distribution location has at the same time also moved to other ‘locations‘, such as Social Network Sites. SNS, as well as UGC´s, are not merely a place of networking and mass communication but are becoming increasingly important as marketing platforms [1]. The relevance of paratexts can be seen particularly strikingly by the example of movie trailers. These short commercial movies, which were originally used to lure the audience back into movie theaters [21], are characterized as paratexts and marketing tools with their own specific dynamic [22,23]. Besides promoting upcoming movies, trailers, as paratexts offer reading instructions, and as Dusi [24; p. 154] emphasizes, “they provide potential audiences with an early understanding of the film’s genre and theme, and open a cognitive and affective challenge linked to a viewer’s curiosity about the film”. Trailers are an ephemeral feature in the context of comprehensive marketing strategies [25; p. 32], which take on different (paratextual) functions. Thus, Williams [26; p. 5] emphasized that teasers and trailers form a look forward moment, which is vital for the audience to *address and engage actively with a text* “or allow us immediately to decide that we will never watch this film” [25; p. 34]. They open opportunities to *identify with the material shown* and to enter the (narrative as well as fictional) world of the advertised movie.

As such, with a view to (marketing-)paratexts and their preparatory functions [27], not only the engagement with the text/movie is predominant in the analysis of cinematic narratives. Ultimately, it is essential to understand how it becomes possible for meanings to be formed by audiences before text/movie reception, by recieving and interpreting companion texts [10; p. 1]. In this context, it should also be considered that paratexts are “not only the ad but also the add. […] After all, if someone is moved to watch a certain movie by a trailer, that trailer will need to have done more than insist that the film be watched, rather, it will need to have constructed a series of meanings, themes, and/or characters to which the audience responds” [11; pp. 308–309].

The focus of scientific work on paratexts is on different questions, which analyze the relevance, the developments, and transformations of this concept, for instance within the framework of the production of meaning [12,13]. Here, the concept of expectations certainly acts as a key to understanding the functionality of contemporary (blockbuster) cinema and of paratexts [26; p. 2]. As such paratexts work and function in a similar way to advertising, “to sell and brand a product” [3; p. 27]. They serve primarily to generate and sustain a need for the final product/text but in return hold a promise, a potential counter value [28; p. 90]. Gray thus also writes that “[p]aratexts matter” (2016, p. 33). Not only because they (can) mark the first contact with a text. Instead, they represent the starting point of all further developments, because “paratexts will either greet us, encouraging us to come in, or they will turn us away, insisting that we are not welcome, that the text exists for others instead” [25; p. 33].

In the context of movie marketing, it is not only intended to generate interest and hype but also to open and establish (emotional) connections and significance for recipients. Movies are not just reducible to the moment of their reception. Instead, the movie experience represents a specific point in a comprehensive appropriation process [29; p. 147]. To do so, movie-industry invest considerable, cost-intensive efforts, including beyond traditional marketing strategies: Viral and buzz marketing campaigns are initiated. They are active on Social Network Sites, they produce and distribute production diaries, are in close contact with fans [16], and distribute movie-related information on specially established websites. All these activities are ultimately associated with creating a relationship with audiences. They intend to arouse interest and curiosity, and to make commercial use of all these efforts; in other words, to get people to buy a ticket and to draw them into movie theaters. This process is as much an open-ended as uncertain one, which Gray thus also terms an “act of faith” [3; p. 24]. In their various formats, be they teasers, trailers, posters, or even Social Media posts, marketing-paratexts focus on hypothetical audiences. As Kernan [21; p. 3] succeeded in demonstrating in her study of trailers, the hypothetical spectator can be identified and reconstructed “within trailer texts themselves”. In this sense, such paratexts, in their dual function as marketing tools and their relevance in the production of meaning, can be regarded “as important signals reflecting the media industry's own conceptualization of the nature, composition, and interests of audiences” [9; p. 99].

## Marketing-paratexts, transmedial storytelling, and commodification of emotions

Paratexts are the “outskirts” [16; p. 33] of each text, which both represent the first point of contact and are also the start of the narrative and its interpretative appropriation. They are of relevance before the text, but also concerning further interpretative engagement and textual developments. “While we still (sometimes) go to the cinema, it [the movie and its paratexts] now surround us everywhere” [30; p. 7].

Instructive theoretical insights related to the functionality of paratexts can be found, e.g., in Jenkins [31,32] and his studies on Convergence Culture. By convergence, Jenkins refers to “the flow of content across multiple media platforms, the cooperation between multiple media industries, and the migratory behavior of media audiences who would go almost anywhere in search of the kinds of entertainment experiences they wanted” [31; p. 2]. In Convergence Culture, the boundaries between audiences, industry, texts, content, interpretations and brand messages become blurred and “the promotion of a media product is collapsed into the product itself”, as Brookey & Westerfelhaus [15; p. 23] have shown using the DVD and its accompanying materials as an example.

“In this emerging media system, what might traditionally be understood as media producers and consumers are transformed into participants who are expected to interact with each other according to a new set of rules which none of us fully understands. […] Convergence occurs within the brains of individual consumers. Yet, each of us constructs our own personal mythology from bits and fragments of information we have extracted from the ongoing flow of media around us and transformed into resources through which we make sense of our everyday lives” [31; p. 3].

In this constellation, which Jenkins analyses as features of affective economics [31; p. 61, 33–36], altered media synergy effects are produced by technological developments, and expanded intratextual relations are evoked. At the same time, affective and emotional features also gain in meaning. Not only are recipients described as “active, *emotionally engaged* and socially networked” [31; p. 20]. Emotions themselves are transferred into capitalist exploitation contexts, commercialized and transformed into products [37,38].

In empirical and theoretical studies about paratextual phenomena, such as those created and distributed for marketing purposes, reference is also repeatedly made to these emotional facets that are inherent to them and their relevance in connection with appropriation and interpretation processes is described [22,39–41]. Concerning the paratextual framing of texts/movies an evocation of emotions is of significance, since “they give consumers the resources to talk about the subject at hand and the tools to interpret its meaning” [9,42; p. 76]. Taking movie trailers as an example, Bleicher [43; p. 254, 44; p. 226] described powerfully that “trailers [function] through *physically intense*, *emotional and surprising stimuli*”. Garde-Hansen & Gorton [45] emphasize the relevance of the emotional engagement of recipients and their significance in the interpretative appropriation, which also occurs above all through paratexts. “Emotions aren't free-floating feelings but rather embodied neural affective processes; that is, they exist empirically as part of our mind/brain”. In this manner, “the relationship […] is seen as a complicated social, psychological, and physiological web” [42]. In this sense, emotions can be understood as “a mental state of readiness that arises from cognitive appraisals of events or thoughts; has a phenomenological tone, is accompanied by physiological processes; is often expressed physically […]; and may result in specific actions to affirm or cope with the emotion” [40; p. 184, 46].

In affective economics, emotions are not only understood as components of individual motivation structures. Instead, emotions are transformed into products and are used as merchandised feelings (‘emodities’) [47]. Those ‘emodities’ are designed, taken up and exploited in the sense of performativity and economic rationality in different cultural and social contexts [37,38,48–50]. Affective economics also includes the aesthetic and emotional capacities of recipients, which are reified and transferred to economic exploitation contexts [1,51]. “The company invites the audiences inside the brand company” [31; p. 20]; emotional affectedness is transformed into a functional component of the ‘storyfication’ of a text, and operates to create “awareness [,which] means creating demand, and demand cannot easily be contained in the formal spaces of trailers, teasers, and marketing paratexts” [52; p. 122]. The purpose of emotional affectedness as a component of paratextual materials is to develop a story *that is subjectively regarded as relevant and significant*. “The emotions are a serious opportunity to get in touch with consumers. And best of all, emotion is an unlimited resource. It's always there–waiting to be tapped with new ideas, new inspirations, and new experiences” [31; p. 70, 53]. Precisely in connection with movies and the paratextual frames that accompany them, this is demonstrated, with the interplay between text and paratext engendering “emotion machines” or “machines for cinematically-feeling-products” [54; p. 141, 55].

Marketing-paratexts have significant meaning with a view to the preparation and establishment of a text. Today, movie-studios take great care to establish a paratextual flow of information and to construct and/or confirm emotional ties, and at the same time involve recipients in the corresponding processes, to let them participate in the development process of a movie [56; p. 533], and to ultimately inspire them to engage with the movies. “In addition to online activities, studios are developing ways in which to deepen their relationship with consumers through strategies based on simple CRM […] systems” [57]. Here, the concept of parasocial interaction and relationship might be helpful to understand the significance of relationship between audiences and industry and the relevance of continuously developing it [58]. Parasocial interaction/relationship is a concept initially developed by Horton and Wohl [59]. It refers to the reaction of a media user to media performers so that the media user perceives the performer as an intimate interlocutor. The underlying assumption is that media producers and recipients behave online similarly to face-to-face situations: Producers offer the recipients the illusion of personal contact through direct address and behavior directed at him. The recipient can respond to this communication offers by moving away from the purely observant position and is actively reacting and actively involved in the process [60; p. 24]. In this sense, Social Network Sites can be characterized as components of a network-compatible ecosystem [61], with various forms of transmedial storytelling in combination with parasocial relationship formation being realized via different platforms and with the use of paratextual materials, “with each medium making distinctive contributions to our understanding of the world, a more integrated approach to franchise development than models based on urtexts and ancillary products” [4; p. 102, 31,62,63].

In the empirical study of marketing-paratexts, investigations looking to include such activities on Social Network Sites and analyzing the functionalities and relevance of different data hardly played a role as yet [9,64]. However, this is precisely where several developments are taking place. The present study contributes to the empirical research of marketing-paratexts on SNS. Taking the example of the activities of different movie-studios, the orientation, and functionality of paratextual frames on Social Network Sites are examined. The following questions guided the research:

1. Of interest was to find out what kind of paratextual (marketing) endeavors on Social Network Sites can be reconstructed and the relevance they have in the process of meaning production?
2. The second goal was to explore and to identify the functions of such paratextual activities, and
3. thirdly, which role emotional affectedness adopts in different paratextual materials and how they are shaped.

Following Meyers [9; p. 99] and Kernan [21], this paper focuses on the hypothetical audiences constructed and embedded in the discursive spaces of Social Network Sites. The article analyzes mechanisms and modes of operation of tweets and feeds in the paratextual accompaniment of movie productions. In the study, I am therefore not concerned with the reception experiences of the users, but primarily I seek to reconstruct the modes of functioning of paratexts, using the example of Twitter-Feeds.

In the following, the results of this empirical investigation are presented. The focal points are the results of qualitative analysis. Therefore the activities of movie-studios were analyzed in the context of their (paratextual) social media activities for six movies, and their Twitter feeds. Subsequently, the subject of the study and Twitter as a Social Network Site will be presented, as well as the method (Grounded Theory) used in analysing the material will be described. Following this, the results of the study will be presented. The article concludes with a summary of conclusions.

## Paratexts and social network sites: An investigation of Twitter-Feeds

Social Network Sites focus on connectivity, networking and mass communication, with user activities on SNS being described as “random chatter, the online underbelly of mass opinions *where collective emotions are formed and where quick-lived trends wax and wane in the public eye*” [1; p. 69, emphasis added, 65; p. 114]. By the example of Twitter it can be shown that the activity of “tweeting has […] multiple meanings, from sending instant short messages to creating a live stream of instant opinions” [1; p. 68]. At the same time, SNS are gaining also importance in the context of movie marketing, where paratexts can be found.

### Twitter, tweets and feed(s) as the subject of research

As a microblogging service, Twitter allows the dissemination of brief content limited to 140/ 280 characters. Tweets can be simple text messages, but also comprise combinations of text, external links, images, videos, GIFs, etc. The interplay between many tweets characterizes an (engineered) flow of information [1; p. 69]. *Twitter* “was launched in 2006, and broke into the mainstream in 2008–09, when accounts and media attention grew exponentially” [65; p. 116]. Approximately 330 million users used the service every month in 2017. The daily volume of tweets sent comprised around 500 million in the same year, with approx. 80% of activities being disseminated via a smartphone or other mobile end devices, and using specific applications (apps). Here, users select other *Twitter-*Accounts that they ‘follow in their stream’; just as they, themselves can be ‘followed’ in their activities. As a result of the structure and a limited number of characters of its content in particular, but also through the systematic setting-in-relation, such as via the #hashtag system or the @reply-function, *Twitter* highlights links and connections, disseminates information and makes it possible to organize comments or contributions in a more general context.

*Twitter* is also used as a tool in movie marketing and as a place where paratexts can be found. As a Social Network Site Twitter offers numerous opportunities for disseminating and distributing information, while at the same time involving its users, enabling them to make their contributions, and allowing them to become, what Bruns labeled, *produsers* [66]. Tweets on *Twitter* are partial fragments, the sense of which can only be developed into a full topic in context and interplay with other *Twitter* users and the total of individual tweets.

In this study six movies and their feeds, in full scope, were analyzed. The sample consists of the movies *Sully* (2016) (@sullymovie) [https://twitter.com/sullymovie?lang=en], *Rogue One*: *A Star Wars Story* (2016) (@starwars) [https://twitter.com/starwars?lang=en], *Finding Dory* (2015) (@findingdory) [https://twitter.com/findingdory?lang=en], *The Conjuring 2* (2015) (@theconjuring) [https://twitter.com/theconjuring?lang=en], *Batman vs*. *Superman* (2016) (@batmanvsuperman) [https://twitter.com/batmanvsuperman?lang=en] and *The Shallows* (2016) (@shallowsmovie) [https://twitter.com/shallowsmovie?lang=en]. The movie corpus originated from a larger sample of another investigation, which used statistical data and methods and examined the activities of production studios on various Social Network Sites (Facebook, Instagram, Twitter) for the 200 most financially successful films of the years 2015 and 2016. However, the results of this preceding research will not be discussed in more detail here. The selection of movies for the present qualitative study was based rudimentarily on the procedure of theoretical sampling. Theoretical sampling is the process of data collection aimed at generating theory, during which the researcher collects, encodes and analyses his data in parallel and decides which data are to be collected next. The process of data collection is controlled by the emerging theory [67, p. 147].

In the course of the research movie-feeds were included step by step, tweets coded, codes evolved to concepts, categories developed, working-hypotheses data-supported refined, discarded, revised or extended. The successive expansion of the sample–including movies of different genres–however, resulted primarily from the research questions and the respective guiding research interests in learning to understand how paratexts are used and work in movie feeds. Movies from the years 2015 and 2016 were the movie corpus [from the previous study], but it was not determined from the beginning, which cases were to be surveyed for the qualitative study [67, p. 56]. The selection of cases was based on a heterogeneous foundation. The movie-sample was not fixed from the outset but was constantly extended against the background of the research question and the iterative orientation of the research process. Both case and theory-related considerations in connection with theoretical sensitivity played a role here. However, the iterative coding process was especially intended to provide new insights. With the help of comparisons and contrasting, for example by including films of different genres, new and expanded perspectives were to be opened. In the selection of films and film genres it was considered whether they would help to understand the phenomenon of paratexts in movie-feeds investigated here. Within the framework of colloquia, method, and research workshops, the material was processed, coded, categorized, and results discussed by several coders. The paper draws on core results from the evaluation for all movies. Approximately 4,000 tweets were coded and fed into the evaluation and interpretation process. The terms and conditions of Twitter have been complied.

### The empirical analysis of tweets and feeds via Grounded Theory Methodology (GTM)

The study focused on an investigation of the functionality of tweets and the relevance of paratextual materials for meaning production. Grounded Theory, according to the approach developed by Strauss and Corbin [67], was used. The choice of Grounded Theory as an evaluation and interpretation strategy is based on the comprehensive offer of coding and development of the material in stages made by the method. Grounded Theory is not only widely used, but can also be characterized as an open and adaptable procedure. Grounded Theory offers a variety of strict rules for the evaluation of (complex) data, such as online content [68]. The focal point of Grounded Theory is/are the phenomenon/phenomena that are investigated. The method offers different coding steps and is designed as an iterative method. The goal is “to reduce, summarize, and condense [the research phenomenon] to its core” [69; p. 179]. With the aid of different coding steps (open, axial, and selective), the materials analyzed here were broken open, codes, concepts, and categories developed. Technical aids and similar computer programs (MaxQDA) were used for this purpose. Four of the six movie-feeds examined have a defined start and end point. These feeds were fully captured for the analysis. For the two other feeds the time periods [several months] during which the films studied were relevant and feed activities and corresponding materialities were focussed on these films, were fully included in the analysis. The feeds were mirrored [the ones with a defined start and end point were fully mirrored; the other two included the period of activity for one movie] to allow further offline editing. The mirrored pages were then transformed into an editable document (.rtf) to be able for using them in QDA-software. The evaluation of tweets was carried out in chronological order. Tweets were therefore added to the evaluation and coding process in ascending order according to the date of their release. Nevertheless, the coding process, especially in axial and selective coding, was not carried out exclusively using technical aids and structuring tools [70].

The coding and annotation of the data and the grouping and development of connections between codes, concepts, and categories were arranged in a network-like data structure and permanently further developed. In addition to In-Vivo-Codes, coding was also based on asking theory-generating questions [How and What is it all about? Which phenomenon is addressed? Who and which persons/actors are involved? What roles do they play? How is it interacted, how and in which way are aspects of the phenomenon presented or not thematized, etc.?]. The main task was to develop those individual components into nodes of a network. The procedure was designed as follows: In open coding, the material was viewed, tweets were serialized, and the material concerning its content was 'broken open'. Here, the goal was to identify the content used in tweets, as well as related information systems. This comprised

- information on how tweets are structured, and whom or what they address,
- how tweets refer to content, and how different information is interconnected during this process.
- Furthermore, temporal sequences were identified, i.e., when and at what point specific information and media content were distributed.
- Equally, the objective was to find out what role emotions/emotional affectedness take on in tweets, what information/information systems are linked to them, how emotional addressing operates and to what they refer.

References were identified, provided with descriptions, designations or (typifying) expressions (codes), which were subsequently condensed into concepts. Coding also included visual means, such as images/GIFs or videos, which were integrated during the process of coding to investigate their functionality and to study their interplay with other tweet components (text). Within the scope of research into visual means, the GTM offers wide-ranging opportunities for developing theoretical findings in connection with rarely explored, visual subjects/practices. The focus here was on the conceptualization of image types [71], which were developed systematically with the aid of the Grounded Theory coding method. Image types encompass recurring image motifs that are identified based on categorical differentiations [72; p. 197], i.e. they comprise a bundling of “all image motifs with a consistent content-related meaning and [differ] in terms of content from other image types” [71; p. 170]. In reference to Konecki [73], who assumes a “grammar of visual narrations” [73; p. 131], visual means were transcribed, textually ‘translated' and coded, considering different reference levels.

As well as visuals means, coding was also conducted on texts. Texts were content-related broken open and coded. Above all, subdimensions and relationships between codes were carved out and further developed into concepts. These concepts were then grouped, set in relation to each other and condensed, in connection to be able to describe (sub-)categories. The production of image descriptions, particularly in memos, emerged as being of relevance [73]. Through memos, access is opened to different levels of an image: the a) inner context of a picture, b) display of practice, c) an interpretation of the picture's content as well as d) an understanding of the outer context of the picture [73; pp. 144ff.]. Thus, the focus is not only on a textual translation of motifs, situations or practices but far more a reconstruction of the grammar of one/several image/images. In this sense, “[m]ultislice imagining […should be understood as] a tool to adjust the mind of the researcher in connecting any specific features of the events with generic processes that should be theoretically elaborated” [73; p. 146]. In the evaluation, motifs were (temporarily) grouped and (tentatively) categorized regarding similarities, differences, and contrasts. As soon as a new motifs were ‘discovered', previous images/motifs and their categorical assignments were revised, compared, assigned, and also re-coded [72]. In turn, in axial coding, the identified concepts and subcategories and the relations between them were continuously further developed. This also included the incorporation and carving out of connections between e.g. text and images/image types, which was focused on reconstructing the functionality of the medium (tweet) and the interplay of its components (texts, image types). Thus, a reconstruction was possible of how developed concepts interplay, i.e., how for example the linking of image/text functions, according to what temporal logic the information dissemination is designed and structured via feeds, and what functions tweets assume in the paratextual flow of information. Thus, increasing abstractions could be achieved, and more general categories could be developed, described, and successively filled with content. In selective coding, in turn, categories were set in relation, and contrasts and reciprocal sets of conditions were developed. With increasing condensation and abstraction, a coherent depiction of the phenomenon was developed step by step.

## Results

The purpose of the study was to identify materials used in tweets and to reconstruct their (paratextual) functions. The following sections compile central results from the evaluation. The argumentation is structured as follows: Initially, an overview is given of which materials are used in tweets, and the activity level of the feeds studied is presented. Then, the reconstructed functions of tweets are presented. Finally, results are compiled and discussed.

### Movies, paratexts and their development over time in movie-feeds on Twitter

**Fig 1:**
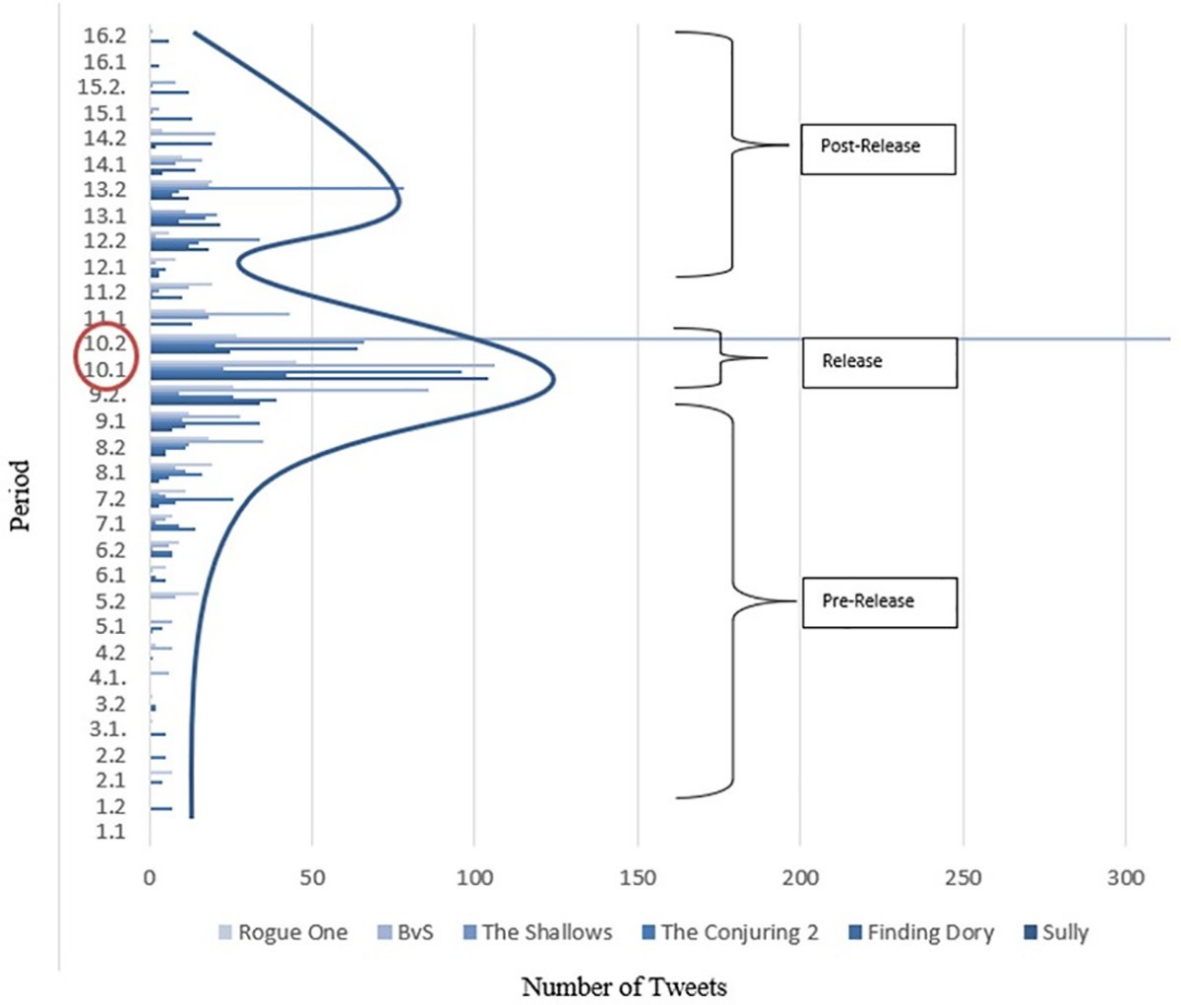
Progression of Twitter activities and ideal-typical progression (study sample).

Tweets (usually) operate as carriers of multiple information, consisting of several components, such as texts, visuals (images/GIFs/videos) and catchwording of contributions with hashtags. Hashtags are “a feature that enable[s] users to group posts together by topic articulating certain words or phrases” [1; p. 71]. Equally, links were identified to previews, making-of´s, sneaks, production diaries, production notes, contributions, and interviews with and by actors involved, the production crew and tv-spots, merchandising, teasers, trailers, posters and contributions that include recipients in different processes. A further realization arising from the evaluation comprises the characterization of the respective activity progressions of these feeds (Fig 1).

The actions of all feeds were standardized concerning the release date to guarantee comparability. The release dates of all movies are the reference points– 10.1 and 10.2 in this case. The periods before and after are shown in months. The first numerical value refers to the months, while the second value defines a compilation period [e.g. 10.1 = the first 15 days of the reference month, 10.2 = the remaining days of the reference month]. Moreover, the activities before and after the release were classified according to different phases: pre-release, release, and post-release. Here, it is evident that an ideal progression form can be described across all feeds. While the level of activity slowly increases during the pre-release phase, especially intensifying around specific cumulation points (e.g. the release of teasers, trailers, posters), the activities during the release phase, usually 2–3 weeks beforehand, increase significantly. In the post-release phase, activities decrease considerably but then rise again with a second-high point (Digital-Release, DVD, Blu-Ray). However, the activity peaks of the second phase are never reached again.

Concerning the functions of tweets, different alignments were identified. The following presentation is based on the categories and relationships between them obtained during the analysis: first, the dissemination of information and specific knowledge fragments, with different tweet-content being aligned towards establishing a text/movie, its content, and components essential to the narrative, as well as presenting and accompanying the emergence and development process. Second, a participative function is linked to this. It is primarily aimed at opportunities for involving recipients, with different opportunities for participation being offered, and users being involved in a related process. Third, tweets and the contents they feature stand out by an affective-emotional side, which comprises both unique characteristics of a movie, its genre and features of a story as an intended ‘affectedness' of the recipients.

### Information and the dissemination of knowledge

Information and background knowledge are the supporting foundations of all feeds examined. The feeds are used to disseminate background knowledge about a movie and its development process. The associated flow of information is aimed at and differentiated concerning the above-described phases of the production process (pre-, release- and post-release phases). In the pre-release period, different materials are used to disseminate production-related content and to establish step-by-step the core topics of a movie. During the phase of the release, these are supplemented by information on qualitative features, particularly with recourse to qualitative evaluations by third parties as well as network-compatible evaluation systems, and relate to the evocation of consumption needs. In turn, in the pre-release phase, the developed images and the reception experiences are taken up, and textual supplements and related appropriation opportunities are offered.

#### Pre-release phase: Information and the creation of an image

The content disseminated in the pre-release phase comprises diverse materials and focuses on different aspects of a movies production. In combination with texts, this also included (links to) short videos, teasers, and trailers and behind-the-scenes material, production diaries, background information on the movie characters, interviews and production notes as well as Images and GIFs with scenes from the movie. The process of content dissemination is organized by the production studio responsible, the directors, but also, in particular, the actors, who take up facets of production, the narrative and share these. Tweets contain and link to different aspects of production and operate similarly to an *everyday companion*. They offer opportunities to participate in an otherwise inaccessible process. Background information focuses above all on the different stages of production, which are taken up, e.g., in set-reports or in production diaries, which present the backgrounds and challenges of creative work [74]. Tweets are used in the sense of representation and as a means of creating an impression. For example, they refer to technological aspects and the creative challenges in this process. The activities in the feeds are first aligned to disseminating such production-related information, often in connection with those involved “documenting […the] creative processes and producing reflexive narratives about their industrial practices” [16; p. 75]. Second, tweets are used to refer to the content of a movie, whereby the construction and establishment *of one or more core topics* are of relevance. This comprises above all the narrative features of a text/movie, which are taken up regularly, but which are also sharpened around specific culmination points such as the release of teasers and trailers, which offer a first look and visual, acoustic and plot-related contact with the advertised movie. In an interplay between texts and visual means and in a time sequence of several months, narrative images and framework features of a story are introduced. Tweets open prospective opportunities and pave the way for recipients to the (not yet) narrated events. They do not specify, but they do show what type a text/movie is concerned or relates to anything that pre-existed, such as prequels/sequels/spin-offs. In an interplay of text and visuals, the components of a narrative are introduced. This creates predictability (genre, actors, directors and style) but also refers to the uncertainties and secrets of a story. The interplay between different materials in this phase and how the core topics are established, will now be described using an example from the feed of the movie *The Shallows (2016)* [@theshallows]:

*The Shallows* tells the story of its protagonist, who is attacked by a great white shark while surfing in a secluded bay. In the pre-release phase, the activities of the feed are aligned to introducing the core-topics. Core-topics were categorized as a ‘(subtle) threat’ and as a ‘fight for survival’. The establishment of the core topics occurs as a procedural activity. Here, both texts and visual data are of relevance, as is the hashtag [#FeartheShallows] used in each post during this phase. Via this hashtag, tweets and their content are catch-phrased and rendered assignable. Users can also access them themselves, use them in posts and “producing heterogeneous aggregates of brand-related content that also operate in relation to the advertising text” [75; p. 174]. Hashtags are a search term and a tool for organizing information and as metadata are a “social resource for building relationships and communities” [76; p. 214]. They open up possibilities for classifying and assigning tweets in connection with image and text posts. Furthermore, however, the texts used, and the visual means are of importance.

The basic thematic components [(subtle) threat (see the example tweets: Tweet1, and Tweet2, and Tweet3) and fight for survival (see the example tweets: Tweet4, and Tweet5, and Tweet6)] are taken up and presented as features of the narrative in a combination of texts, visuals and with variations of text and image continuously. Text and image(s) function as narrative components, which refer to different facets of the movie, describing and visually illustrating them.

With a view to the reconstruction of the functionality of tweet components within the framework of the establishment of movie-related basic thematic components, codes were identified, categories worked out, and relationships between these and the basic thematic components developed. In this context, the development of the functional interplay of different tweet components, i.e., text and image, proved to be challenging. To make the procedure more tangible, I present some results and intermediate steps from the evaluation, by introducing coding, concept and categorial examples (Table 1). While in the first phase monomodal coding of text and image was the focus, in axial coding especially the multimodal correspondences between text and image were examined to analyze the audiovisual interplay of tweet components, i.e., how they correspond to each other. The objective was to reach step by step a higher level of abstraction and thus to be able to arrive at and describe the storyline and specific patterns of a pre-receptive online discourse.

**Table 1:** Codes, concepts, and categories and the establishment of core topics. T = Text, IT = Image Type.

(Example-)Codes	Concepts	(multimodal) categories
• Surfing, leisure, and sports • Attack while surfing • The attack is the beginning • Inevitable attack as narrative facet • Question: What happens after the attack?	features of the course of the narrative (T)	(subtle) threat
• Shark (symbol) • Animal as a danger • Observation, point of view shark • Moment of attack = fast • Moment of attack = unforeseeable • attack occurs suddenly • Camera perspectives/cinematographic stylistic elements	attack as an unavoidable but unforeseeable story element’ (IT)	
• Location: Bay, Beach, Ocean • Abandoned place • Alone/ loneliness • No one can hear you scream Wherever she goes, the shark will follow • No help • Indication, shark otherwise in depth now in Shallows	the setting of the narrative (T)	
• Abandoned place • Paradise/Paradise lost • coast, sea, • deserted beach • Get lost (in the Ocean)/Nature and Danger • cloudy/opaque water	nature at first look idyllic, but unpredictable and dangerous (IT)	
• “Shark sees you. Shark breathes you. Shark hunts you.” • warnings, sharks as a real danger • when swimming /at the beach • You/ Can you; Are you able to…; Its on you…	addressing of recipients (T)	
• Attack and danger • anxiety, fear, panic • Surfing and shark silhouette • Invisible Threat/Indications of Danger	the protagonist' and its ‚situation’ (T)	Fight for survival
• The ocean as danger/ Danger comes from the depth • wave metaphor/Wave, Shark, comes closer • underwater/ above water (Perspective) • Shark Outlines/ Hints at shark • the dorsal fin/symbol • Something is in the water • Danger lurks beneath the surface. • There is something in the water	subtle references to the animal/the threat’ (IT)	
• Nature is savage • Nature = innocent and danger • Nature unaffectedness • human incursion • Nature as a challenge • human invasion into habitat	nature as something unpredictable and challenging’ (T)	
• violation • offensive • Setback and wound through the attack • The shark can smell a drop of blood	vulnerability’ (IT)	
• defence against shark • defence against the forces of nature • The fight against nature in the form of sharks • Human vs. great white. • battle for survival	Defence (IT)	
• Fight against the sea • the opacity of the water surface • Fight against sharks • Being on your own/ Alone in this fight • Encouragement/Outsmart. Outwill. Outlast.	the fight against nature’(IT)	
• You don't see sharks, but warnings about them • A Vacation you won't forget • Shark genre and movie as representatives thereof • Can you survive?	addressing of recipients (IT)	

In this context, text posts function largely as taglines; they are a source for describing the kind of movie being advertised. Such texts inform potential audiences of a movie's content, and they are a facet of a branding process, with texts always being strategically used as verbal design elements in combination with visual means [77,78]. Text contributions were coded and categorized as notifiers and attractors of attention. For example, texts comprise different coded references to the ‘setting of the narrative’ [“Beware the rising tide. #FearTheShallows” (tweet: 04/25/2016)], the ‘protagonist and its situation’ [“No-one can hear you scream. #FearTheShallows” (05/03/2016)]. Furthermore, ‘nature is described as something unpredictable and challenging’ [“Nature is savage #FearTheShallows” (04/07/2016)], specifically demonstrated by the subtle threat from the shark, just as the references to ‘features of the course of the narrative’ [“The fight is for your flesh. #FearTheShallows” (04/04/2016)] and the addressing of recipients [“How fast can you swim? #FearTheShallows” (03/08/2016] were coded. The texts/taglines used to refer to the different facets of the story and point to these.

The words and taglines are designed to evoke interest in an upcoming film by creating representations of a movie. There are similarities but also differences from slogans/taglines of standard film posters. There are similarities but also differences with slogans/taglines of standard movie posters [78]. In contrast to posters, the texts/taglines on SNS are more diverse and complex. While taglines on movie posters *reinforce a movie’s image in one short phrase*, texts/taglines on SNS are more varied in their design. In different tweets, not only one characteristic slogan is used, but to a far greater extent a wealth of varying text posts, which touch on and refer to different facets/features of a text/movie. Texts/taglines operate and function as a variant of branding slogans, which as memorable phrases sum up the premise and the tone of a brand, a product, or a movie. Such texts refer to and characterize the facets of a story, which, however only function in interplay with visual means. Texts name and refer to different aspects and content, which are, at the same time, visually taken up and presented. Different image types contextualize and situate the narrative, and refer, visualize and construct in connection with texts/taglines the core topics. For the (subtle) threat, for example, image types were reconstructed that describe ‘nature at first look as idyllic, but on second glance, it becomes unpredictable and dangerous', or as ‚subtle references to the animal/the threat', or which seek to establish the ‘attack as an unavoidable but unforeseeable story element'. Other image types focus on the protagonists and their fight for survival, with image types and motifs of ‘vulnerability', for example, as well as of ‘defence' and ‘the fight against nature' have been identified. Image types do not just bundle motifs with a similar or the same statement, but at the same time also refer to different focuses and the narrative components of the story. However, texts/taglines and visuals work in combination. Texts refer to a specific facet, mark this and seek to bundle the premises and the tone of a movie into characteristic statements, which are then also shown visually. As a result, a text-visual-representation of the movie is created, an interplay that is not dissimilar to that of movie posters, but which is not limited to just one motif and one tagline. Instead, it allows a combination of large numbers of texts and themes and the creation of impressions and the establishment of images of a movie over a more extended period. In the mix of text and image, the facets and characteristics of the story are presented, which are repeatedly taken up in different variations, marked as being of importance and are anchored through the process of telling (text) and showing (image). Tweet components and their interplay function as narrative and visual components, which design the essential parts of a story as a movie-related heuristic. An orientation field is built up, the crucial elements of a story are established, with the maintenance of a balance between the familiar and the innovative is of importance “so as to simultaneously generate a reference point within the audiences understanding of cinema and the curiosity which will make them want to consume” [79; p. 132].

#### Release phase and information function

With the transition into the release phase, the activities in all examined feeds increase considerably concerning the dissemination of information. Activities of the feeds and the content released to focus on the one hand on the confirmation of the core topic(s) of a movie that was developed during the pre-release phase, but also seek to evoke consumption needs with a view to the release date, by adding qualitative characteristics. The materials used here expand the image of a text/movie, with, as well as narrative and creative-artistic facets, quality features of a narrative and options for the reception being presented above all. Reviews, user critiques, etc. are taken up in tweets, reference is made to network-compatible statements and evaluation systems, and corresponding evaluations are shown in text posts and visually. Similarly, information that takes up quality aspects of a movie, with facets on the review of the movie by professional as well as user critics being of relevance. On the one hand, reviews by individual critics and their core statements are shared in combination with images and links to complete reviews, and on the other hand, references to summarized qualitative reviews are disseminated. This information is taken up by tweets in all examined feeds, with reviews on the site www.rottentomatoes.com, the ‘Tomatometer score’ and the Certified-Fresh Seal being picked up on in particular and being used as information carriers for statements on quality (see example tweets: Tweet7, and Tweet8; other movie-examples [Sully: Tweet9 and Tweet10; The Conjuring 2: Tweet11 and Tweet12). The Tomatometer score represents the percentage of professional critic reviews that are positive for a given movie or television show. Certified Fresh means that a movie has a Tomatometer score of 75% and higher and a required minimum number of reviews.

Once again, tweets operate as complex information carriers. In an interplay of text and image, the special narrative features of a text/film are in turn presented, which are supplemented by qualitative statements, i.e. for example by declarations regarding the dramaturgical and directional qualities of a text/film, reference is made to individual reception opportunities and an evocation of reception needs is intended. In turn, the texts/taglines used are of importance. The texts are used to refer to special textual and narrative features of a story on the one hand, while on the other seeking to initiate reception processes by referring to the unique qualities of a narrative. [“There's something in the water. See Blake Lively in #TheShallows, only in theatres. http://sonypictures.us/ShallowsTixTW”, tweet: 07/09/2016), “Grab on for your life. See the movie thrilling critics and audiences TODAY. #TheShallows” http://sonypictures.us/ShallowsTixTW”, tweet: 07/09/2016); “Can you outswim what's in the water? Dip your toes in #TheShallows, now ‘Certified Fresh’ on @RottenTomatoes”, tweet: 06/13/2016)]. Once more, texts are marked by hashtags and expanded by links, e.g. to websites for purchasing tickets. This also applies to the visual means used. With the help of the above-reconstructed image types and motifs of the pre-release phase, the essential components of the story are shown once more (subtle threat and fight for survival), which as illustrated by the example given in the example tweets above, are expanded by e.g. the ‘Certified Fresh Seal' as an expression of a network-compatible evaluation system by critics as evidence of quality.

#### Post-release phase and information function

The activities in the feeds examined as well as the contend disseminated reduces considerably in the days and weeks after the release. Although occasional tweets are still posted, these refer in particular to the fact that the movie can be viewed in cinemas in connection with qualitative references. The activities of the feeds increase again before the release of the digital, DVD and Blu-Ray versions of a movie. The focus is on the date of release and the opportunities offered through a consumption process of being able to see the movie repeatedly as well as the bonus materials and further background information. With a view to the coding activities and functions of tweets, these assume intermediary roles, which refer above all to opportunities for textual expansions, which are offered in bonus materials, DVDs/Blu-Rays and/ digital versions of a movie. Related matters are above all of importance to specific groups of recipients, such as fans, with opportunities for textual expansion being highlighted and addressed accordingly. These can in medias res change the course of interpretations after encountering a text or guide audiences toward specific interpretations. Gray & Lotz [80; p. 134] argue that such texts are “not the mere add-ons or ‘secondary’ entities”. To a far greater extent, for those who know them, they become a facet of the text itself, they are a part of their version and understanding of the text. “Many industry-created paratexts try to set limits for interpretation […] inviting the audiences to look at the program in a certain way” [80; p. 134]. As a part of these industrial activities, the tweets studied refer to such textual expansions and the related, individual options to expand meanings of a text.

### Participation and interaction purposes

As well as the dissemination of information, (background-)knowledge and the establishment and confirmation of the core topic(s) of a movie in connection with references to reception and textual expansions, secondly, different participative purposes in the use of feeds and tweets were coded. This resulted from the mode of operation of *Twitter*, the dissemination of content, the localization of topics and themes via #hashtags and the unpredictable activities of recipients as well as opportunities for them to write their postings, comment on others or to retweet them. Social Network Sites such as *Twitter*, *Facebook*, *Instagram* and even *Snapchat*, are facilities for networked communicative and interactive activeness and a place for platformed sociality [1], with the related communicative and interactive possibilities are also a facet of the (paratextual) frameworks of movie productions on SNS and are used in different ways. Tweets are, as described above, information carriers, which are used from production companies as well as moviemakers to disseminate various pieces of information, but also seek to evoke interest and curiosity for a media product, e.g., movies. The tweet flow is conceptualized as a live-stream of instant, short and short-lived messages and opinions, “a stream that supposedly taps a real-time undercurrent of opinions and gut feelings” [1; p. 78]. As well as such essential communicative functions, tweets in the feeds studied are used within the scope of initiation and to maintain communicative processes and to offer opportunities for participation within the range of textual and interpretative accompaniment, which were described and reconstructed in different connections. The related communicative activity is conducted in two categorically different formats, which are both aligned to involving recipients in communicative processes, creating visibility and developing (controlled) external effects, while at the same time, differences were identified concerning their manner of operation. First, this comprises a (1) time-restricted and controlled communicative activity between users, the followers of a feed and those involved in a production–directors and actors, above all–which is intentionally introduced via tweets. Second, participation was of relevance as (2) facets of the textual and interpretative frames with a view to the establishment of specific readings.

Both formats and the related activities were coded in the material in different contexts and feed activities. The former were coded in the context of time-situated events, which were categorized as *Community Events*. In the latter case, specific actors were of importance. Their activities are not temporally bound, and they conduct interactive activities with recipients that are possible at any time, with such actors usually taking on *gatekeeper-functions*. Those *gatekeepers* write their posts and regularly respond to questions from the community.

There are many occasions for this. On the one hand, the communicative exchange in this connection refers to the embedding of events or background knowledge on movies or other developments. On the other hand, such communicative and participative opportunities were reconstructed as features of a permanent contextualization process. As such, contact and interaction opportunities are enabled, and interpretation offers are opened up, which are part of relationship cultivation between the industry, brokered by *gatekeepers*, and audiences, but beyond this also seek to establish bonds with audiences as well as particular readings of a text/movie, which guide viewers toward specific interpretations. Gatekeepers take on different tasks: they enter active communication with users, they contextualize, establish, confirm or negate readings, select and drive an 'editorial line'.

While gatekeepers are permanently involved in different communicative processes with users, and their activities can be interpreted as live communication, Community Events are temporally restricted, structured, and controlled interaction occasions in the feeds. Here, recipients are involved within the scope of such events in temporary, communicative processes. This comprises, e.g., those such as the Question & Answer Events, but also other options for time restricted opportunities to participate. For example, recipients are invited to make creative contributions, can take part in competitions and specific events, such as movie premières, web shows, etc., which open temporally restricted options for participation and occasions for interaction which involve ‘followers’ [81].

Regarding the functionality of such participatory opportunities, they are intensively used to negotiate an intentional and controlled image as well as the embedding of information communicatively, establish specific readings, raising awareness, and building customer loyalty. The resulting relationships are the sum of multiple (parasocial) interaction sequences. On the one hand, the mostly imaginary character of the relationship allows it to be conducted free of obligations and responsibility. On the other hand, what Horton & Wohl [59] once described in the parasocial interaction as one-sided is transformed in various forms of mediated interactions with others, a reciprocal devotion of communicants (other recipients and/or controlled participation and interaction purposes). Interactions are the basis for establishing parasocial relationships; they involve, they form bonds with audiences as well as particular readings and meaning which in turn are also linked to the evocation and variation of emotions and moods. Twitter is used in this context as a communicative tool for interactions, which open up reciprocal accesses to the social reality of the communicants, between recipients, and recipients and producers. They offer the illusion of personal contact through direct address, which resembles a social relationship. These relationships can, with reference to Homrighausen [82], be described as forms of 'mimicry-social relationships', with sociality and interactions being constructed and evolved, but which function as part of competitive structures for attention, curiosity, loyalty, and hype. Such forms of a participation focus on what Caldwell [83; p. 69] has described as an “intertextual commodity”, which is realized in different formats as digital intimacy [84]. Related activities and the options for participation opened via them involve users, and on Social Network Sites, low-threshold opportunities are provided for engaging recipients in corresponding processes. Recipients are involved in an event, and communicative processes are created. Here, content is not only passively retrieved and consumed, but to a far greater extent, followers of the feed are involved in corresponding procedures with their posts, their perspectives and questions, ideas, positions and creative output. “In a participatory culture, members also believe their contributions matter and feel some degree of social connection with one another (at the least, members care about others' opinions of what they have created)” [85; p. xi]. Ultimately, the related activities are also facets of storyfication and storytelling. Recipients experience attention; they are visible and are made visible, are seen and listened to and involved in different processes. At the same time, such events are strategically used, not only “by embracing social media to engage directly with their audience” [85; p. 121], but above all also in order to performatively design textual framing and reframing in communicative and networked events, and ultimately to convince viewers to invest in the labour of reception.

### The production of meaning and the relevance of emotions

Up to this point, it has been shown that in the dissemination of knowledge and (background) information about a movie, as well as in participative formats that focus an involvement of users in communicative and interactive forms, two attributes are named that are characteristic for the framing of a text/movie on a Social Network Site such as Twitter. Options for textual appropriation are opened up, i.e., frames through which recipients can make sense of what a text is or could be. Furthermore, in the reconstruction of the modes of operation of tweets, a third property was identified, which is of relevance in the construction of the field of experience of a movie. The interplay between information, knowledge and participative factors and the underlying materials is intended to produce, in particular, attention, curiosity, excitement, and hype for an advertised text/movie. The data studied, always also feature text-dependent, affective-emotional addressing, which accompanies the binary interplay between promotion and art and the emotional experience of a text/movie, the understanding of the text and its interpretative appropriation. In this context, (marketing) paratexts on SNS also attempt to echo the (affective-emotional) meanings of a movie by constructing it as an emotional experience. Different materials in tweets refer to content and features of a text, they allow participation, involve followers, open up *insights and offer impressions* of textual and emotional qualities/features, and seek to evoke text-dependent psychological and physical excitement. The contents of tweets open opportunities in different facets to experience emotional elements of a text/movie as pre-receptive characteristics. They offer emotional impressions and seek to establish a psychological state with varying quality, duration, and closeness to awareness.

**Fig 2:**
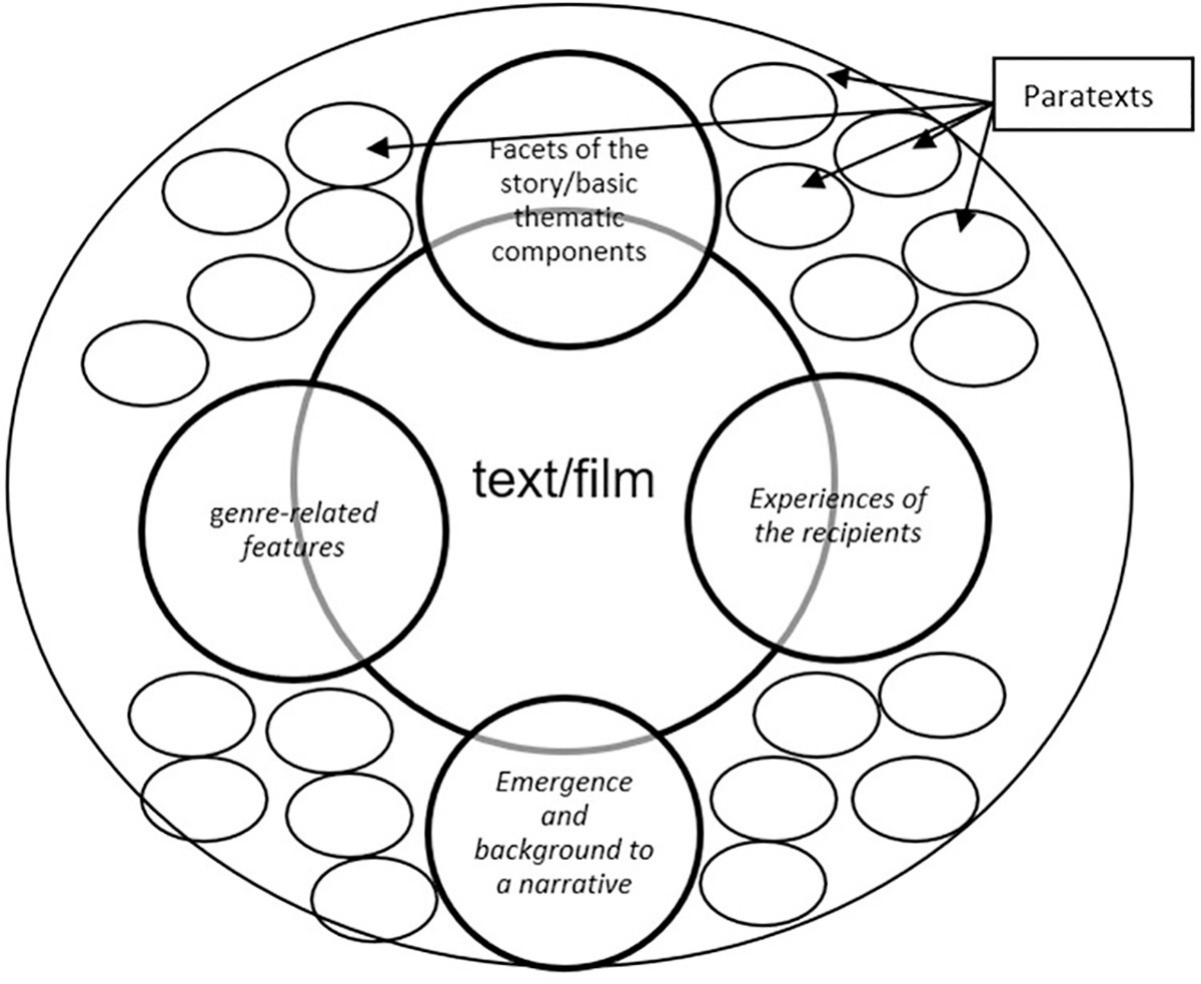
Affective-emotional perimeter.

In this sense, emotions evoked by movies and the cinema are not just a facet of the moment of reception, but also a feature of the framing, (para)textual accompaniment and in the development of dramatic composition of a text/movie on Social Network Sites. Movies open the opportunity to express and experience feelings. These are qualities, which are taken up and presented as facets of paratextual accompaniment. These pre-receptive distinctive features were reconstructed in the material as features of an affective-emotional perimeter (Fig 2), with which the evocation of anticipated emotions and moods is linked in conjunction with different tweet content, and a text/movie with specific emotional foci and affected qualities is constructed and developed. At the or center of the perimeter is the text/movie, which is prepared, introduced and framed with the aid of different (paratextual) materials, which develop the readings of a text as companion pieces.

These also include the construction of textual tonalities, of moods, emotions, as well as affects, which create an emotional field of experience. These accompany and structure pre-receptive appropriation and interpretation processes, just as they are equally oriented, in terms of economic utilization, “to produce an economic effect more swiftly and surely than economics itself means that affect is itself a real condition, an intrinsic variable of the late-capitalist system” [86; p. 45]. They are therefore by no means just an ancillary product, but an essential component of the storyfication of the text on SNS and of its commercial exploitation. Depending on a specific movie and its genre, this comprises different foci in the development of pre-receptive text dramaturgy on SNS, which are produced and addressed via corresponding tweet content. Characteristic textual-emotional features are presented via tweets. For example, comedies are presented as capable of making audiences laugh, or horror films are presented as generating fear and being terrifying, and movies are thus created as emotional commodities. Through accompanying paratextual materials on SNS, such textual features are presented as unique qualities of a text/movie, and they refer to the fact that movies can do something with recipients, that it is a part of their essence to evoke affects, and influence feelings and moods. The associated interplay is complex and will be now be outlined by taking examples from the feed of the movie *The Conjuring 2* [2016, James Wan]: *The Conjuring 2* is the sequel to the movie that appeared in 2013, *The Conjuring* (James Wan), and, like the former, is a horror movie. Horror movies are focused on the evocation of specific emotional states and affects, distinctive features that are also characteristic of the pre-receptive framing of the movie on Twitter. Once again, the interplay between different tweet components, and particularly of the text and visual means, is of significance.

Texts operate, as shown above, as notifiers and function as slogans/tagline texts. Those taglines describe a movie in different facets with the aid of *concise formulations* and refer to a textual range of *pleasures and emotions*. Here, such texts operate in categorically different contexts. For example, they refer to previous text and movie experiences and corresponding qualities (“The first one was a masterpiece, but wait till you see this one #TheConjuring2”, tweet: 04/20/2016]), focus on and name emotional qualities (“the fear is real #TheConjuring2”, tweet: 04/16/2016] or they address recipients in connection with emotion-related distinctive features (“Are you afraid of the dark? #TheConjuring2”, tweet:03/03/2016]). In this case, texts also function as attractors of attention, which present what recipients can expect from a text/movie, how this can be understood, and what qualities and what enjoyment a text/movie can provide. Texts/taglines refer to textual distinctive features and conditions, they intend to attract attention, they contextualize, and they encapsulate textual elements. At the same time, text posts are aligned towards an interplay with visual means and are dependent on them. Here, in the combination of text and image, content and features of a story, in other words, *what* a movie offers, are rendered visually more specific, as is *how* he does this. Tweets refer respectively to specific facets, emphasize a feature, a distinctive characteristic, which is then taken up visually and presented. In an interplay of different materials, it is shown, for example, what type of text a movie is, to which genre it belongs, what story is told, what characters and their relations are of relevance, and also the tonality of a text and the emotional features that are of importance. In doing so, again images/motifs were analyzed in feeds, alongside text posts. In conjunction with the communication of information and knowledge, background information, participative structures also emotional appeals were reconstructed in the feeds in four categorial contexts, which are specified and constructed through text posts, but above all also through visual means. These are:

- The *underlying thematic components*: here, the focus is on textual features–the story, protagonists, antagonists, setting and essential textual elements–which are ‘framed' emotionally and in terms of content and which characterize a text; in other words, states of emotional affectedness are always also intended. They vary according to the film and refer to different focal areas as well as the tonality of a movie.
- *The presentation of the background to the creation of a narrative as a creative and imaginative process*: this comprises insights into the production process and a characterization of these processes as creative and inventive work, with the corresponding materials being used to present the *effort and challenges* involved in a production process, and with the passion and commitment among those involved characterizing and addressing an emotional involvement.
- Textual *experiences of recipients and the visualization of emotional states of affectedness*: on the one hand, textual references to *previous reception experiences that are connected to specific emotional qualities* are taken up, mainly concerning predecessor films and spin-offs. On the other hand, *reception experiences of the film being advertised are presented in connection with specific emotional qualities*, *such as in user review*s and also in visual posts, which *take up and visualize* reactions to a film among viewers, e.g. during test screenings, etc.
- g*enre-related features in connection with emotional qualities*: in tweets, a genre’s characteristic features are furthermore taken up as text-dependent facets in association with emotional appeals and corresponding attributes. Tweets shape the path of events and make this predictable through genre-specific references; they seek to incite anticipatory actions and to evoke textual anticipations.

In the sense of the procedure of multislice imaging, introduced by Konecki [73], images were broken up in the coding process, memos were written and step-by-step image types developed from different motifs in an iteratively designed process. These image types were then arranged with a view to their foci, and further developed (Table 2). Image types and the motifs that form their basis are used intensively in the pre-receptive framing of a text/movie on SNS to visualize the grammar of a text and the possible meanings by constructing and framing a movie as an emotional experience in different dimensions.

**Table 2:** Image types and perimeter dimensions.

		Image types	Examples for image motifs
**Perimeter dimensions**	Facets of the story/essential thematic components	• Emotional and affective resonance	Motifs seek to show the display and the visual *co-experiencing* of affective and emotional qualities and to evoke physical and psychological excitement.
		• Basic thematic components	Motifs are aligned to the establishment of narrative features, the core topics, of a movie in connection with specific emotional qualities.
		• Figure constellation and identification	Motifs present figures and figure constellations, visualize and characterize the roles of protagonists and antagonists. Motifs seek to establish text-related identification in connection with essential narrative components, with motifs show the challenges, for example, that protagonists face.
	Experiences of the recipients	• Revitalization	Motifs of revitalization refer to previous text experiences and emotional and affect-related qualities. Topics are focused on movie-related knowledge and the pre-receptive skills of recipients in connection with emotional qualities, particularly concerning prequels, sequels, and spin-offs.
		• Collectivity and reception experience	Motifs of collectivity *visualize the reception experience*, which is designed as experience in community with others.
	Reference to movie- or genre-related features	• Memory type and elements of a genre	Motifs of memory create textual references to other, previous texts/movies and revitalize reception experiences in connection with specific emotional qualities of a genre.
		• Points of focus	Motifs contextualize a movie as a genre representative and focus on similar features, distinctive characteristics, and abilities in connection with specific qualities.
	Emergence and background to a narrative	• Process and action type	Motifs describe a movie production and the course of production as a process, which is characterized by different production stages and which is connected to various challenges and efforts.
		• Professionalism and commitment	Motifs focus the participants in a production process, particularly the actors, directors, and producers, and characterize these as such who understand their profession and who work towards completing the production in a committed way.
		• Creativity	Motifs of creativity focus the production of a movie that is built upon the creative achievements of individuals involved. At the same time, images visualize that creative challenges can only be overcome in an interplay of those involved.

As well as stills, this occurs in tweets, particularly in the form of moving image material, in individual cases in videos, but particularly in images of the Graphics Interchange Format (GIF). Taking the example of the image-type of emotional and affective resonance, I will show how visual materials are used on SNS, and their functionality is explained in the context of emotional production of meaning. The following two example tweets present two of the GIF posts used and the image type to which they were assigned during the analysis. Tweet 1 was published on 04/16/2016, and Tweet 2 on 03/19/2016, in other words, three and four months before release, respectively. The two tweets each contain a textual reference alongside the GIF image sequence [Tweet13: “The fear is real”; Tweet14: “Just when you think it's over…”] and the Hashtag #Theconjuring2.

In both tweets, emotional qualities are visualized, and recipients are addressed. In the scope of two movie scenes, reference is made to a movie-specific and genre-specific experience of fear. In the image sequence of the first tweet, the facial expression of a protagonist (a girl)–a movement of the eyes–is shown, which in the GIF image sequence is directed towards an invisible outside subject. The GIF documents an emotional quality, with a dramaturgical construct being created in the movement visualization and facial expression features and a fear reaction being shown. In connection with the tagline of the tweet [“The fear is real”], this emphasizes an emotional quality of the movie. The visual material of the second tweet is designed in a similar manner, with here, not only a distinctive emotional characteristic being visualized, but a physical affect reaction being intended. Here, recourse is also made to cinematographic means. The GIF image sequence comprises three changes of perspective in total. At the center of attention is a protagonist, who is focusing on something in a rigid, unbelieving way. Through a shift in perspective, this becomes somewhat more visible; one sees a wall on which crosses that have been hung up are moving. A part of the room is in darkness and cannot be seen. From precisely this area, without warning, the silhouette of a body and two hands approaches, which reach out to grab the girl. In the third change of perspective, the terrified drawing back of the protagonist can be seen, but the threatening figure has disappeared. *The impact of the GIF* is oriented to the unforeseeable moment–the sudden appearance of this figure–with the dramaturgy of the image having a dual design.

On the one hand, the image sequence focuses on a disconcerting scene (the girl observes how the crosses move on a wall), which takes on a new quality through the emergence of a new figure. The tagline "Just when you think it's over…" refers to the specific and dramaturgical attributes of the film/the film series and the scene is portrayed as being able to disconcert and terrify. Scenes in the GIF and the tagline here refer to distinctive characteristics and abilities of the movie. After all, neither the protagonist nor the audience can detect the frightening element before its sudden appearance. The GIF focuses on a disconcerting scene and intends to produce a substantial affect impact, while visualizing a quality of the horror movie of being able to terrify and shock, and presents this ability by conducting the effect itself visually.

In both GIFs, the circumstances of the scenes cannot be seen; they are presented in a decontextualized manner. Primarily, visualizations of textual qualities in combination with an affective excitement reaction, are in the foreground. The power of the images is not in what they represent, but in how they do it. This does not occur statically via an individual image or indicated or textually described, but to a greater extent, in the image sequence of the GIF, specific (cinematic) movement sequences are opened up, which refer to specific affective-emotional qualities. The images intend to generate physiological and psychological excitement through a visual stimulus. Here, textual conditions are presented that are oriented towards emotional overwhelming. Visual means in tweets open insights and new information regarding the inner view of the imaginary world and the narrative, with which recipients can engage in adapting textual and emotional meaning offers. Visual means in tweets open perspectives. They present the thematic emphases, along with protagonists, antagonists, figure constellations and plot related features of the story, and always also offer movie-related emotional qualities, in this example e.g. fear, terror or disquiet; qualities that are distinctive characteristic features for the horror genre, and movies that are consumed and that can generate fascination *by being frightening*, *threatening and shocking*, with a visual-emotional story being told, and the movie being introduced as an emotional experience through tweets. Ultimately, connected to all these activities is the intention of establishing a resonant relationship, by sparking interest and curiosity for engaging with a text/movie and finally to also exploit all these production and marketing efforts financially. In other words: to convince people to buy a ticket for the movie and to draw them into the theaters. “In applying its knowledge of feelings, the movie industry constructs feelings per this knowledge” [54; p. 140f.]. Through corresponding materials in tweets, the initiation of a consumption process is intended, in which alongside the emotional attractiveness of a movie, text-specific interpretation and meaning offers are supplied, which are taken up by recipients and must be adapted, and which contribute meaningfully to the text/movie as an artistic object.

## Conclusions

Using the above empirical investigation, the meaning and relevance of marketing paratextual companion phenomena on a Social Network Site were examined. The essay focused this phenomenon from different perspectives, analyzing the paratextual activities of movie-studios using the example of six Twitter feeds. Tweets are used to disseminate information and knowledge; they make participative offers whereby emotions are a focal point and are intended in tweets.

Regarding paratexts on SNS, these companion texts are not only intended to generate attention but are also used to nurture the relationships between producers and recipients. This relationship is aimed at demand, loyalty, and engagement as well as emotional ties and their consolidation. As such, Twitter is used as a tool in movie marketing, which movie makers and producers utilize to open up temporary opportunities for identification. Feeds and tweets operate as information carriers, which take up existing textual references, knowledge and experiences of recipients and offer individual consolidation and appropriation opportunities. They are also used to integrate recipients into these processes, providing them with opportunities to address and engage with the advertised text over a long period. Individual tweets operate as fragments in a comprehensive process, which, in their interplay, open up opportunities for sense and meaning production.

Several essential insights result from this investigation, which are also vital in the understanding of movie audiences and the production of meaning. The research illustrates that movie-studios are actively involved on Social Network Sites and fall back on paratextual materials as part of their activities, which place themselves around the medium as an interpretative perimeter. At the same time, the associated activities and efforts can be described as characteristics of a complex interactive process. In this, audiences are actively involved to establish a relationship. However, commercial interests are also inextricably linked with this. As ads, the marketing-paratexts studied here attempt to evoke reception and consumption needs; at the same time, they are also a part of the textual appropriation and are relevant for the commitment, interest, and curiosity as facets of the interpretative introductions to a text. Paratextual “materials in movie and television become [central] to the culture that surrounds the movie and show” [3; p. 309]. At the same time, marketing-paratexts sell a product, are an ad for a text. They intend to make commercial use of all these production and marketing efforts at the box office, to overcome the act of faith by establishing meanings, emotional connections in a complicated resonant relationship. However, they also add something to texts; they evoke interest, curiosity, and hype, disseminate information and are aimed at the establishment of a tie between a movie, its paratexts and audiences in the interplay of all these activities.

Paratexts on SNS *tell and prepare* the story before it hits the movie theater. As such, a battle for attention in the ephemeral information stream on Social Network Sites has become apparent. Although the movie is still at the center of all these activities, the conditions of production, distribution, marketing, and reception have changed fundamentally. Marketing processes are incorporated into Social Network Sites, in a comprehensive communicative process, and are a facet of the DNA of a text/movie, which develops and enriches this in equal measure. As companion texts marketing-paratexts on SNS are intended to evoke and maintain attention, expectations, hype, and curiosity.

Moreover, the concept of paratexts undergoes “important transformations, starting with the fact, that many of them are arranged in the same horizon of the consumption of the text itself” [29; p. 147]. This is seen vividly in the relevance and importance given to paratexts by movie- and production studios in the marketing process, especially on Social Network Sites. *Twitter* is, on the one hand, a discovery mechanism. On the other, however, *Twitter* is also a tool, where individual remarks condense as fragments of an extensive discourse, where public statements result “through new syndication technologies […] into a process of citation, syndication […] accumulation, compilation and finally aggregation” [87; p. 125]. *Twitter* operates as a partial aspect of a synaesthetic network, where impressions are intended. Tweets and the paratexts disseminated there add meanings and prepare the text reception; they demand participation, comments, likes, and retweets, to address and engage with a text and its facets. The paratexts, studied here, offer types of reading, touch on the what and how of a cinematic narrative and generate possible meanings and consumption motives in the interplay with the audience, while at the same time integrating recipients into these processes, giving them the resources to talk about the subject and the tools to interpret its meaning'.
